# Hemolytic anemia caused by aortic flap and inversion of felt strip after ascending aorta replacement

**DOI:** 10.1186/s13019-016-0520-1

**Published:** 2016-08-02

**Authors:** Masayuki Sakaguchi, Tamaki Takano

**Affiliations:** 1 Department of Cardiovascular Surgery, Suwa Red Cross Hospital, 5-11-50 Kogando-ri, Suwa City, Nagano 392-8510 Japan; 2 Department of Cardiovascular Surgery, Nagano Red Cross Hospital, 5-22-1 Wakasato, Nagano City, Nagano 380-8582 Japan

**Keywords:** Aortic dissection, Hemolytic anemia, Aorta replacement

## Abstract

**Backgrounds:**

Hemolysis related to a kinked prosthetic graft or inner felt strip is a very rare complication after aortic surgery. We describe herein a case of hemolytic anemia that developed due to aortic flap of the dissection and inversion of an inner felt strip that was applied at the proximal anastomosis of a replaced ascending aorta 10 years previously.

**Case presentation:**

A 74-year-old woman presented with consistent hemolytic anemia 10 years after replacement of the ascending aorta to treat Stanford type A acute aortic dissection. The cause of hemolysis was attributed to mechanical injury of red blood cells at a site of stenosis caused by aortic flap of the dissection and inversion of the felt strip used for the proximal anastomosis. Repeated resection of the strip and graft replacement of the ascending aorta resolved this problem.

**Conclusions:**

We considered that blood flow disrupted by a jet of blood at the site of the proximal inner felt strip was the cause of severe hemolysis, we describe rare hemolytic anemia at the site of aortic flap and inverted felt strip after replacement of the ascending aorta.

## Background

In general, hemolysis related to a kinked prosthetic graft or inner felt strip is a very rare complication after aortic surgery. We describe herein a case of hemolytic anemia that developed due to aortic flap of the dissection and inversion of an inner felt strip that was applied at the proximal anastomosis of a replaced ascending aorta 10 years previously.

## Case presentation

A 74-year-old woman underwent emergency replacement of the ascending aorta because of Stanford type A acute aortic dissection at another hospital. The entry of the Debakey type I aortic dissection was located in the middle of the ascending aorta, and extended distally to the distal abdominal aorta. The brachiocephalic artery and left common iliac artery were also dissected. Graft replacement of the ascending aorta was done, which were used by Teflon felt strip and GRF glue to reinforce the anastomosis of the dissection aortic wall. The post-operative course was unremarkable except for leg paresis that might have been due to spinal infarction caused by the dissection.

**Fig. 1:**
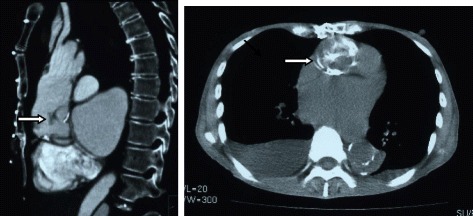
Chest CT scan before second surgery. Severe stenosis at proximal anastomosis of inner felt strip (*arrow, right*) and aortic intimal flap (*arrow, left*) (*right*, plain CT; *left*, enhanced CT)

**Fig. 2:**
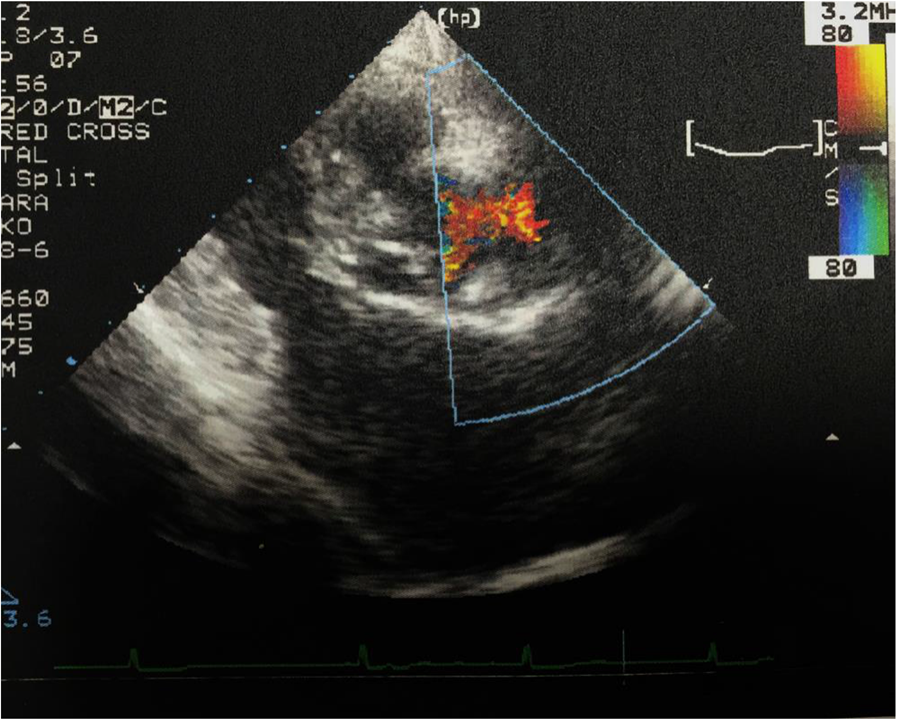
Transthoracic echodardiography. Color-flow doppler shows mosaic pattern in the felt strip on the ascending aorta

She was diagnosed with a small cerebral infarction, jaundice and anemia 10 years later and admitted to our hospital. Blood tests revealed severe hemolytic anemia. The laboratory results were Hb, 7.3 g/dl; T. BIL, 4.06 mg/dl; and LDH, 3020 IU/l. Microscopy revealed fragmented red blood cells. No stigmata were found that could be associated with TTP or ITP. CT revealed severe stenosis at the proximal anastomosis of the inner felt strip and aortic intimal flap (Fig. 1), which was one of two layers of Teflon felt strips positioned using the sandwich technique to reinforce the dissecting aortic wall. The pressure gradient at the site was not measured because it was impossible to insert a catheter. Transthoracic echocardiography (TTE) showed mosaic pattern in the felt flap of the ascending aorta although pressure gradient could not be measured (Fig. 2). We thus considered that the cause of the hemolytic anemia was mechanical injury caused by disrupted blood flow and repaired the proximal anastomosis.

**Fig. 3:**
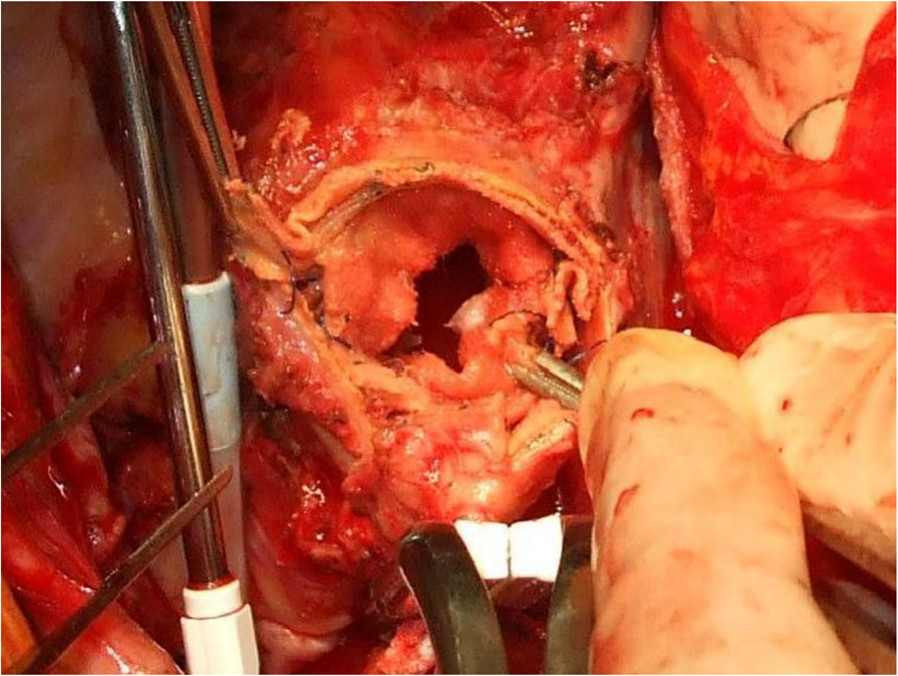
Intraoperative findings. Stenotic aortic lumen of ascending aorta caused by circumferentially inverted internal felt strip

**Fig. 4:**
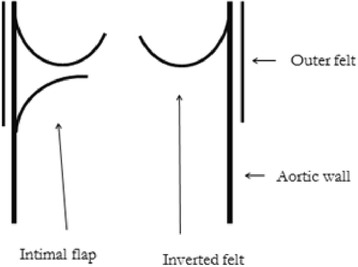
Intraoperative findings. We identified local dissection and intimal flap of the proximal site of ascending aorta at noncuspid valve site

The patient underwent a second operation. ECC was established via a median sternotomy with a cannula at the distal site of the ascending aortic graft and a two-staged venous cannula in the right atrium. A thrill was felt at the ascending aorta, and we cross-clamped the distal site-of the ascending aorta. We opened the prosthetic graft to explore the proximal anastomosis and found that the inner felt strip was turned up and that it had reduced the diameter of the inner lumen (Fig. 3). We also identified local dissection and intimal flap of the proximal site of ascending aorta at non cusupid valve site (Fig. 4). The proximal site of the graft, local dissection of the ascending aorta and the Teflon strips were removed, and the ascending aortic was replaced once again with a 22-mm graft that was directly sutured to the previous graft at the distal site of the ascending aorta. The patient was quite easily weaned from ECC and hemostasis achieved without difficulty.

The postoperative course was uneventful, and no fragmented red blood cells or progression of anemia was observed. The serum LDH and T.BIL levels gradually returned to normal.

## Discussion

Hemolysis after cardiac surgery is a rare but recognized complication, especially as perivalvular leakage after prosthetic valve replacement. However, hemolysis related to an inner felt strip or a kinked prosthetic graft is very rare [1–7]. We described hemolytic anemia that developed at the site of an inverted Teflon felt strip after ascending aortic replacement. Teflon felt strips have been widely applied during cardiovascular surgery to reinforce anastomoses. However, Izumi et al. [1] and others [2–4] have described several complications arising from surgery to treat acute aortic dissection. The dissecting wall in our patient was reinforced using the sandwich technique with two layers of inner and outer Teflon felt strips. The inner strip had become inverted and caused stenosis of the internal lumen. Thus, the collision of red blood cells with the felt strip and severe aortic stenosis at the site because of the inversion might have contributed to the hemolysis. However, whether the hemolysis could be attributed to the blood flow being disrupted by a jet of blood at the site of constriction or the reversed inner felt, or both, remained unclear. The post-operative laboratory findings of our patient remained normal for 10 years after the first operation. This is the first report to date of hemolysis arising at such a late point after surgery. The use of GRF glue to reinforce the anastomosis of the dissecting aortic wall for the first operation might be some relationship. Because late adverse event such as pseudoaneurysm and re-dissection have been reported to result from the use of GRF glue [8]. So this event might have been more inverted for the inner Teflon felt strips.

Imaging studies are required for diagnosis and, except for a systolic ejection murmur, laboratory findings were compatible with those of red cell fragmentation syndrome. In our and other patients [1, 2, 4], CT (especially three-dimensional reconstructed scans) and magnetic resonance angiography were beneficial [3, 4]. TTE was also helpful to clarify stenosis at the felt strop in our case. Mosaic signal indicating turbulence was detected with TTE as shown in previous papers [2–4]. Mosaic pattern in TTE with red cell fragmentation syndrome are considered essential to determine the existence of stenosis and hemolysis at the felt strip. Reoperation should be performed if the patient presented with persistent anemia due to mechanical red cell fragmentation.

To prevent complications arising in such patients, the internal felt strip should be as narrow as possible, and its most proximal portion should be sutured to prevent it from curling up. Three-dimensional reconstructed CT scans and transesophageal echocardiography can help to identify anastomotic stenosis that develop after surgery to treat aortic dissection using Teflon felt strips for reinforcement.

## Conclusion

We described rare hemolytic anemia that developed at the site of an inverted felt strip 10 years after replacing the ascending aorta to treat aortic dissection. Patients who have undergone aortic surgery using an inner felt strip and GRF glue should be carefully followed up over the long term.

## Abbreviations

CT, computed tomography; ECC, extracorporeal circulation; GRF, geratin-resorcin formalin; Hb, hemoglobin; ITP, idiopathic thrombocytopenic purpura; LDH, lactate dehydrogenase; T.Bil, total bilirubin; TTP, thrombotic thrombocytopenic purpura
